# Epigenetic silencing of HOPX is critically involved in aggressive phenotypes and patient prognosis in papillary thyroid cancer

**DOI:** 10.18632/oncotarget.27187

**Published:** 2019-10-15

**Authors:** Yosuke Ooizumi, Hiroshi Katoh, Mitsuo Yokota, Masahiko Watanabe, Keishi Yamashita

**Affiliations:** ^1^ Department of Surgery, Kitasato University Hospital, Kanagawa, Japan; ^2^ Breast and Endocrine Surgery, Kitasato University Hospital, Kanagawa, Japan; ^3^ Division of Advanced Surgical Oncology, Research and Development Center for New Medical Frontiers, Kitasato University Hospital, Kanagawa, Japan

**Keywords:** HOPX, papillary thyroid cancer, epigenetic silencing, promoter methylation

## Abstract

HOPX is involved in multiple organ development and acts as a tumor suppressor in various cancers. Epigenetic silencing of HOPX via its promoter methylation has been shown frequent and cancer-specific in human cancers. The proliferation of thyroid cancer cells and cancer progression are strongly influenced by epigenetic alterations as well as genetic changes. Papillary thyroid cancer (PTC) comprises the vast majority of thyroid cancers and exhibits slow progression. However, ~10% of patients still show disease recurrence and refractoriness to treatment. Accordingly, it is important approach to research epigenetic mechanisms in PTC progression to find useful biomarkers. Here, we aimed to seek into the roles and clinical impact of epigenetic silencing of HOPX in PTC.

The promoter methylation of *HOPX* was observed in five of six human thyroid cancer cell lines. Down-regulation of HOPX was seen in three cell lines including PTC line K1, and demethylating agents restored HOPX expression. The promoter methylation was observed with high sensitivity and specificity in human PTC tissues. *HOPX* promoter methylation independently predicted disease recurrence in PTC patients. Epigenetic silencing of HOPX was associated with Ki-67 expression. Of note, *HOPX* promoter methylation was dramatically associated with worse prognosis especially in patients with stage I PTC. Forced HOPX expression suppressed cell proliferation, invasive activities, and anchorage-independent growth *in vitro*.

*HOPX* promoter methylation is frequent and cancer-specific event, leading to aggressive phenotype in PTC. Epigenetic silencing of HOPX may be a clue to tackle cancer progression and have clinical impact as a novel biomarker in PTC.

## INTRODUCTION

Papillary thyroid cancer (PTC) is one of the most prevalent malignancies and comprises the majority of all thyroid cancers [1]. Although the most of PTC exhibit slow disease progression and increasing ultrasonography screening has contributed to early detection of PTC and providing more treatment options for patients, ~10% of patients are still diagnosed with advanced or metastatic disease [2].

Accumulation of genetic alterations and epigenetic gene modifications are one of the hallmarks of cancer [3]. Cancer emerges as a result of such epigenetic changes or genetic abnormalities [4]. In differentiated thyroid cancers, several genetic alterations such as *BRAF^V600E^* mutation, *RET/PTC* or *PAX8/PPARγ* translocation and *RAS* mutation are thought involved in carcinogenesis in thyroid follicular cells, and further genetic change drives stepwise dedifferentiation of cancer cells [5]. A growing body of evidences have demonstrated that complicated mechanisms of carcinogenesis and cancer progression cannot be solved by genetic alterations alone, but also involve epigenetic modifications such as DNA methylation, histone modifications, and microRNA expression [6]. The differentiation and proliferation of thyroid cancer cells are strongly affected by epigenetic alterations, results in cancer progression [7]. Accordingly, it is important approach to seek into epigenetic mechanisms in thyroid cancer progression in order to identify a clinically available biomarker.

*HOPX* (GeneBank accession number NT 022853), also known as *HOP*, *NECC1*, *LAGY* or *OB1*, belongs to a homeobox gene family, and is involved in gene transcription. Three spliced transcript variants (*HOPX*-α, -β, and -γ) encode a same protein, which is a transcriptional corepressor that is essential for the normal development of the mammalian organs [8]. HOPX expression is ubiquitous in a variety of normal tissues, but is attenuated in malignant tissues including choriocarcinoma, lung, uterine endometrial, breast, and gastrointestinal cancers [9–17]. The mechanisms of HOPX inactivation is essentially caused by promoter DNA methylation in endometrial, esophageal, gastric, colorectal, and breast cancer. Moreover, enforced HOPX expression inhibited tumor progression, and knockdown of endogenous HOPX restored the tumor aggressiveness by influencing several mechanism of cancer cell activities [12, 18, 19]. These evidences suggest that the *HOPX* plays a role as a tumor suppressor gene.

Using a pharmacological unmasking microarray method, we have identified novel tumor suppressor genes which are epigenetically silenced in a cancer-specific manner [20]. Among them, promoter methylation of *HOPX* is observed very frequently in a cancer-specific manner, and is correlated with worse long-term prognosis in esophageal squamous cell carcinoma [21], gastric cancer [14], colorectal cancer [12], pancreas cancer [15], and breast cancer [13]. We revealed that HOPX plays suppressive roles in tumor angiogenesis, proliferation, or invasion [12]. Despite that epigenetic DNA modifications may be critical events also in thyroid cancer, the clinical importance and the involved mechanisms of HOPX in thyroid cancer has been elusive. In this study, HOPX expression and the DNA promoter methylation status were assessed in human thyroid cancer tissues and cell lines. Here, we particularly focused on the clinical impact of epigenetic silencing of HOPX in PTC that comprises the majority of thyroid cancer.

## RESULTS

### Structure of *HOPX* promoter region

**Figure 1:**
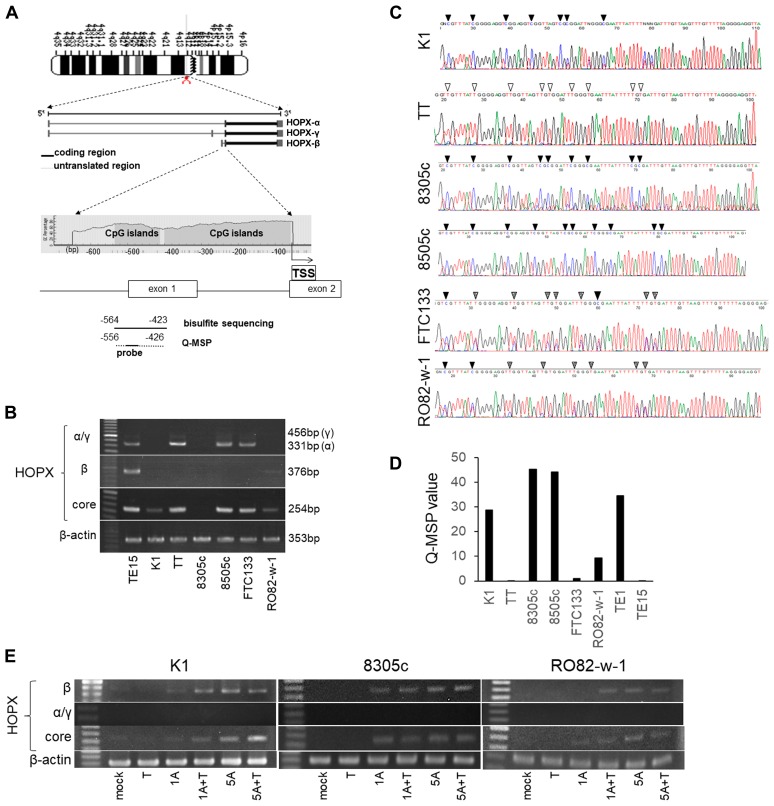
Analysis of promoter DNA methylation of HOPX and its expression in human papillary thyroid cancer (PTC) cell lines. (**A**) Schematic diagram of the genes coding 3 spliced transcript variants of HOPX (the second middle panel) and CpG islands (gray area) in the 5′-flanking region of HOPX (bottom panel). Arrows with dotted lines below the bottom panel indicate the sequences for bisulfite sequencing analysis and Q-MSP. TSS, transcription start site. (**B**) mRNA expression of HOPX variants in thyroid cancer cell lines. TE15 served as a positive control of HOPX-β and core expression. (**C**) Representative results of bisulfited direct sequencing assays in 6 thyroid cancer cell lines. Arrowhead indicates dinucleotide CpG. Filled or unfilled arrowhead indicates methylated or unmethylated CpG site, respectively. Dotted arrowhead means partial methylation. (**D**) Q-MSP values in 6 thyroid cancer cell lines. TE1 and TE15 served as a methylation positive and a negative control, respectively. (**E**) mRNA expression of HOPX-β, -α, -γ, and -core in K1, 8305c and RO82-w-1 cell lines after treatment with a demethylating agent, 5-Aza-dC, in the presence or absence of TSA, a histone deacetylase inhibitor. 1A or 5A, 1 or 5 μM 5-Aza-dC; T, TSA.

The CpG islands of the promoter region of *HOPX* is shown in Figure 1A. *HOPX* has 3 transcript variants, of which only *HOPX*-β harbors promoter CpG islands (promoter B) encompassed by the first exon and intron. All 3 transcript variants share the same open reading frame.

### Promoter methylation of *HOPX*-β critically affects HOPX expression in human papillary thyroid cancer cell line

HOPX expression was initially examined in 6 human thyroid cancer cell lines. PTC cell line K1 and FTC cell line RO82-w-1 express faint *HOPX*-core reflecting very weak *HOPX*-β expression, but did not express *HOPX*-α and -γ. On the other hand, *HOPX*-β mRNA expression was not detected in TT (MTC line), 8305c, 8505c (UTC lines), and FTC-133 (FTC line). *HOPX*-γ expression was not observed in all 6 thyroid cancer cell lines tested. Among them, *HOPX*-core transcript is expressed in TT, 8505c and FTC-133 in concordance with *HOPX*-α transcripts (Figure 1B), suggesting that *HOPX*-α dominantly regulate HOPX expression in TT, 8505c and FTC-133.

Promoter DNA methylation status of *HOPX*-β was tested in 6 human thyroid cancer cell lines by direct sequencing. Promoter region of *HOPX* exhibited complete methylation in cytosine residues of CpG islands in K1, 8305c, and 8505c (Figure 1C). Partial methylation was observed in FTC-133 and RO82-w-1. Promoter methylation was not detected in TT. Indeed, the methylation status of *HOPX* in these cells were confirmed by the methylation levels quantified by Q-MSP (Q-MSP values) (Figure 1D). PTC line K1, UTC lines 8305c and 8505c showed high Q-MSP values than other cell lines.

To postulate that promoter methylation of *HOPX*-β critically affects HOPX expression, cell lines (K1, 8305c, and RO082-w-1) were treated with a demethylating agent 5-Aza-dC alone or in combination with a HDAC inhibitor trichostatin A, as a previous report showing the synergistic effect of 5-Aza-dC and a HDAC inhibitor [22]. Expression of *HOPX*-β and *HOPX*-core but not of *HOPX*-α and γ were restored by 5-Aza-dC treatment with or without trichostatin A in all 3 cell lines (Figure 1E). These findings suggest that the promoter methylation of *HOPX*-β is pivotally involved in *HOPX* silencing in K1 (PTC line), 8305c and RO082-w-1. Here, we focused on patients with PTC that consists the vast majority of thyroid cancer.

### Tumor suppressive activities are increased in PTC cell line overexpressing HOPX

**Figure 2:**
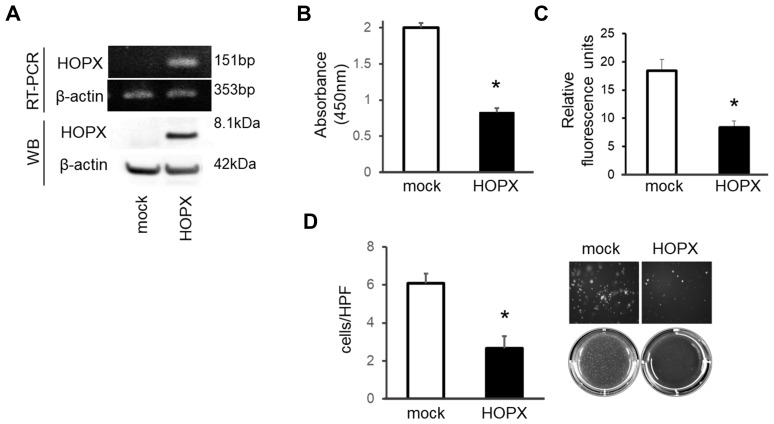
Tumor suppressive activities of HOPX in papillary thyroid cancer cell line. (**A**) HOPX mRNA (top) and protein (bottom) expression in control (mock) and HOPX transfected K1 cells. Western blot (WB) analysis was conducted using a HOPX-specific monoclonal antibody (3D6). (**B**) Proliferation assay was performed for 5 days. Forced HOPX expression significantly suppressed proliferation of K1 cells. Data are expressed as absorbance levels at 450 nm. Experiments were repeated twice in triplicates. *p < 0.05. Error bars, SEM. (**C**) Matrigel invasion assay using a CytoSelect™ 24-Well Cell Invasion Assay. HOPX induction attenuated invasive activities of K1 cells. Cell growth for 24 hours determined by the WST-1 assay was similar (data not shown). Two independent experiments were done in triplicate, and values indicate means ± SEM. *p < 0.05. Error bars, SEM. (**D**) Anchorage-independent colony formation assay was performed using the indicated transfected cells. After 3 weeks of cell culture, colonies were photographed at 100× magnification under a microscope. Colonies were visualized by EtBr and counted. HOPX transfectants exhibited remarkable reduction of colonies compared with mock transfected cells in K1 cells. *p < 0.05.

A construct containing the full-length cDNA of HOPX was built as described in Materials and Methods section. This construct is common to all three transcript variants and is transiently transfected to K1 PTC cells. As shown in Figure 2A, mock transfected K1 cells express very weak HOPX. As expected, enforced HOPX overexpression resulted in increased levels of HOPX mRNA and protein. The mRNA levels in the HOPX transfectants were comparable to those in normal human thyroid tissues (data not shown).

To investigate the effects of HOPX reactivation, several tumor aggressiveness assays were performed. Forced HOPX induction suppressed proliferative ability in K1 cells (Figure 2B). Matrigel invasive activity was significantly attenuated by HOPX expression (Figure 2C). In addition, HOPX transfectants exhibited remarkable reduction of colonies compared to the mock transfectants in an anchorage-independent colony formation assay (Figure 2D). These results indicate that HOPX is pivotally involved in tumor suppressive activities in human PTC.

### Promoter methylation of *HOPX* is frequent and caner-specific in human PTC tissues

**Figure 3:**
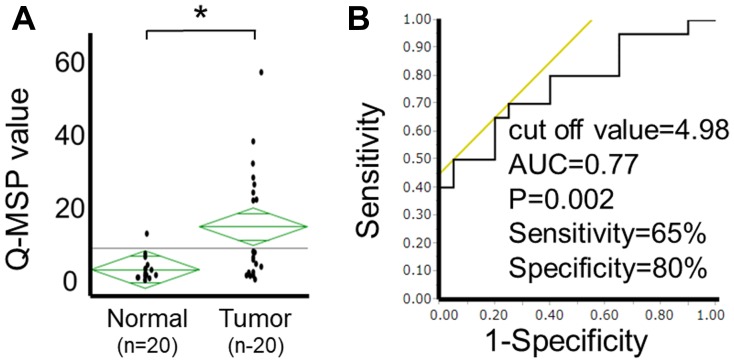
Analysis of *HOPX-β* Q-MSP values in human PTC. (**A**) Scatterplots of *HOPX*-β Q-MSP values of primary tumors and paired normal tissues. *p < 0.01. (**B**) ROC curve of HOPX-β Q-MSP values for distinguishing between malignant and normal samples. Area under the curve (AUC) is 0.77 and represents the accuracy in distinguishing PTC from normal samples in terms of sensitivity and specificity (*p =* 0.002).

To evaluate the clinical relevance of the *HOPX* methylation level, Q-MSP values of PTC tissues were initially assessed in 20 independent patients with PTC. The Q-MSP values of paired normal tissues were also tested. The median Q-MSP value of primary tumor tissues was 7.4, ranging from 0.7 to 57.0. In contrast, the Q-MSP value of paired normal thyroid tissues was significantly lower (median 2.0 (0.4-13.0)) (*p =* 0.002, Figure 3A). A receiver operating characteristic curve (ROC) determined the optimal cutoff value (4.98) for distinguishing between malignant and normal tissues, yielding 65% sensitivity and 80% specificity (Figure 3B).

### *HOPX* promoter DNA hypermethylation predicts worse prognosis in PTC patients

**Figure 4:**
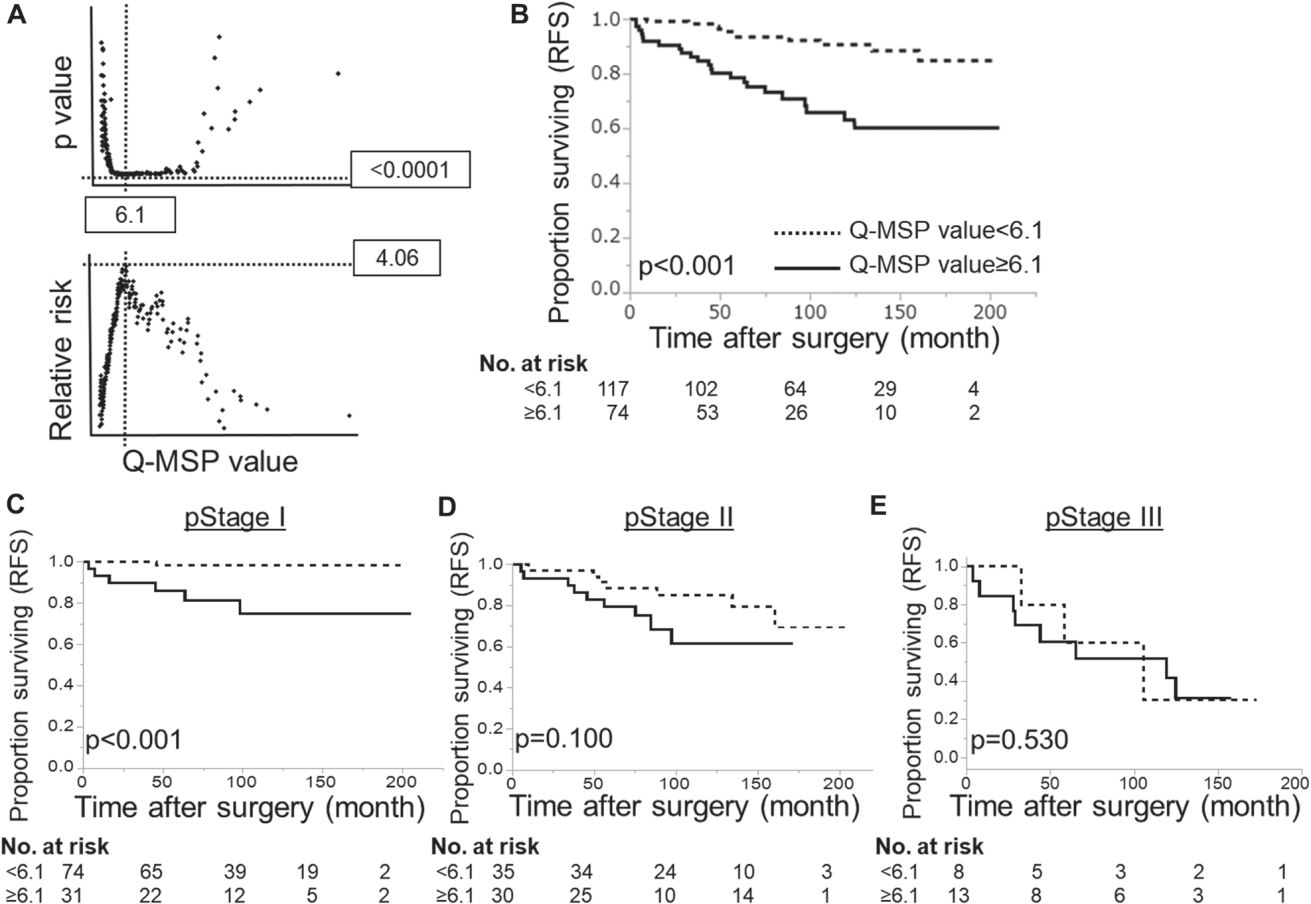
Prognostic implication of HOPX-β Q-MSP values in patients with PTC. (**A**) Log-rank plots were used to determine the optimal cut-off (6.1) of Q-MSP value for the 10-year RFS. (**B**) Kaplan–Meier analysis of 10-year RFS according to Q-MSP values using 6.1 as a cut-off level (*p <* 0.001). (**C**–**E**) Kaplan–Meier analysis of 10-year RFS in each stage according to Q-MSP values (C: stage I, D: stage II, E: stage III). Patients with hypermethylation of HOPX-β exhibited dramatically poor long-term prognosis than the counterpart in pStage I PTC (*p* < 0.001).

Next, *HOPX* Q-MSP values in 191 PTC patients were analyzed in association with long-term prognosis. The optimal cut-off Q-MSP value (6.1) was determined using log-rank plots (Figure 4A), and the following prognostic analyses were performed based on this cut-off value. Kaplan–Meier curves indicate that patients with high Q-MSP values exhibited dramatically worse RFS (10-year RFS=70.3%) compared with their counterparts (10-year RFS=92.3%) (Figure 4B). Patients with high Q-MSP values showed marginally poor overall survival (OS) (Supplementary Figure 1A). A Cox proportional hazard model was employed to perform a multivariable prognostic analysis (Table 1). In univariable analyses, elder age, male gender, tumor size, extrathyroidal extension, pathological lymph node metastasis (pN), higher pStage and high Q-MSP values exhibited significantly poor 10-year RFS among tumor factors (Table 1). Since pT and pstage were confound factors with tumor size and extrathyroidal invasion, these variables were excluded from the multivariable analysis. In the multivariable analysis, high *HOPX* Q-MSP value, elder age, and pN independently predicted worse prognosis.

**Table 1:** Prognostic analysis of clinicopathological variables in 191 patients with PTC

Variables		No. of Pt.	10-year RFS (%)	Univariable analysis*			Multivariable analysis*		
				*p*	HR	95%Cl	*p*	HR	95%Cl
Age	<55	92	92.5	<0.001	0.27	0.11–0.58	0.006	0.33	0.13–0.74
	≥55	98	75.5		3.77	1.73–9.40		3.02	1.35–7.65
Gender	male	32	65.6	0.002	3.50	1.62–7.17	0.442	1.38	0.59–3.06
	female	159	87.4		0.29	0.14–0.62		0.72	0.32–1.70
pStage**	I	105	93.3		reference				
	II	65	78.5	<0.001	3.56	1.52–9.27	n/d	n/d	n/d
	III	21	52.4		11.06	4.35–30.15			
Tumor diameter (cm)	≤1	54	96.3		reference			reference	
	1<, ≤2	53	86.8	<0.001	3.56	0.86-23.89		1.93	0.44–13.30
	2<, ≤4	65	80		5.87	1.62-37.50	0.204	2.49	0.64–16.53
	4≤	19	52.6		18.80	4.84-123.42		4.68	1.04–33.36
Ex***	Ex0	73	94.4		reference			reference	
	Ex1	88	83	<0.001	2.83	1.12–8.61	0.747	1.22	0.43–4.0
	Ex2	30	61.3		7.91	2.93–24.90		1.60	0.48–5.63
pN	pN0	64	98.4		reference			reference	
	pN1a	50	90	<0.001	6.07	0.98–116.16	0.007	3.04	0.6422.1
	pN1b	77	67.5		23.46	4.98–418.96		7.17	1.82–48.47
*HOPX*-β Q-MSP value	Low (<6.1)	117	92.3	<0.001	0.21	0.09–0.45	0.037	0.44	0.2–0.95
	high (≥6.1)	74	70.3		4.68	2.22–10.74		2.26	1.05–5.08

pStage was excluded from the multivariable analysis because the confound factors (tumor diameter, Ex, and pN) were included in the analysis.

^*^Cox proportional hazard model. ^**^accoring to UICC 8th classification. ^***^Ex indicates extrathroidal extension. Ex0, absence of extrathyroidal extension;

Ex1, gross extrathyroidal extension invading only strap muscles (sternohyoid, sternothyroid, or omohyoid muscles); Ex2, gross extrathyroidal extension beyond Ex1.

Abbreviations: HR, hazard ratio; CI, confidence interval; n/d, not determined.

In the dissociative analyses taking stages into account, patients with high Q-MSP values exhibited significantly poor prognosis in stage I PTC (*p <* 0.001, Figure 4C). On the other hand, patients with high Q-MSP values have prognostic association only marginally in stage II PTC (*p =* 0.100, Figure 4D), and no statistical significance in stage III (Figure 4E). Similarly, patients with high Q-MSP values showed poor 10-year overall survival in stage I PTC (Supplementary Figure 1).

### HOPX protein expression closely reflects *HOPX* promoter DNA methylation status, and epigenetic silencing of HOPX promotes cancer cell proliferation in human primary tumor

**Figure 5:**
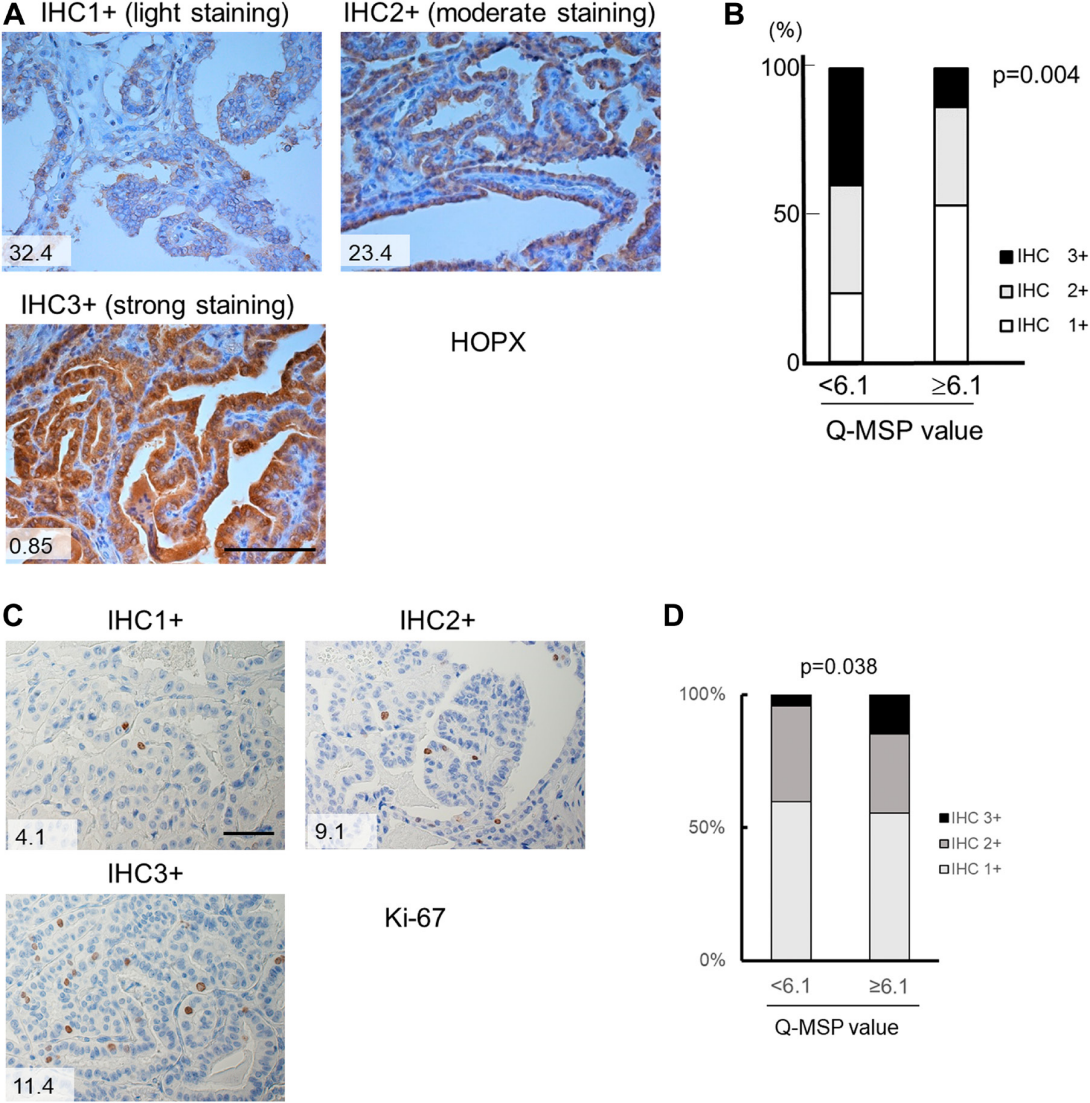
Immunohistochemistry of HOPX and Ki-67 in human primary PTC tissues. (**A**) Representative images from immunohistochemistry of HOPX in primary PTC tissues with light expression (IHC 1+, left upper panel), moderate expression (IHC 2+, right panel) and strong expression (IHC 3+, left bottom panel). Values in the images denote Q-MSP value. Bar, 100 mm. (**B**) Cumulative bar charts indicate that PTC tissues with high Q-MSP significantly exhibit reduced HOPX expression (*p =* 0.004). (**C**) Representative images from immunohistochemistry of Ki-67 in primary PTC tissues with light expression (IHC 1+, left upper panel), moderate expression (IHC 2+, right panel) and strong expression (IHC 3+, left bottom panel). Values in the images denote Q-MSP value. Bar, 50 mm. (**D**) Cumulative bar charts indicate that PTC tissues with high Q-MSP significantly exhibit reduced Ki-67 expression (*p =* 0.038).

Immunohistochemistry confirmed that HOPX protein expression was significantly weak in primary tumor tissues with high Q-MSP values (≥6.1) (*p =* 0.004, Figure 5A and 5B, Table 2). Patient with lower HOPX expression level showed worse prognosis than the counterpart as well although it did not reach statistical significance (Supplementary Figure 3). Accordingly, HOPX Q-MSP value may be more sensitive predictor for long-term prognosis than HOPX immunohistochemical detection. Furthermore, immunohistochemical assay of Ki-67 revealed that Ki-67 expression is significantly higher in patients with HOPX hypermethylation (*p =* 0.038, Figure 5C and 5D, Table 2). These results show that silencing of HOPX by its promoter hypermethylation promotes cancer cell proliferation in PTC. Collectively, epigenetic silencing of HOPX expression caused by the promoter methylation is critically involved in cancer progression and patient prognosis in PTC.

**Table 2:** Correlation of clinicopathological variables with HOPX-β Q-MSP value

Variables		No. of Pt.	HOPX-β Q-MSP value				p^*^	Multivariable analysis
			low (<6.1)		high (≥6.1)			
			No.	%	No.	%		p^**^
Age	<55	41	28	68	13	31	0.297	n/d
	≤55	150	89	59	61	41		
Gender	male	32	11	34	21	66	0.001	0.778
	female	159	106	67	53	33		
pStage	I	105	74	70	31	42	0.007	n/d
	II	65	35	54	30	46		
	III	21	8	38	13	62		
Tumor diameter	≤1	54	40	74	14	26	<0.001	0.016
	1<, ≤2	53	38	72	15	28		
	2<, ≤4	65	35	54	30	46		
	4≤	19	4	21	15	79		
Ex	Ex0	73	56	77	17	23	0.001	0.282
	Ex1	88	48	55	40	46		
	Ex2	30	13	42	18	58		
pN	pN0	64	48	75	16	25	0.017	0.480
	pN1a	50	29	58	21	42		
	pN1b	77	40	52	37	48		
HOPX	IHC 1+	56	25	45	31	55	0.004	n. a.
	IHC 2+	86	55	64	31	36		
	IHC 3+	49	37	76	12	24		
Ki-67	IHC 1+	111	70	63	41	37	0.038	n. a.
	IHC 2+	64	42	66	22	34		
	IHC 3+	16	5	31	11	69		

^*^χ^2^ test. ^**^multivariable logistic regression analysis. n. a., not assesed.

### *HOPX* promoter methylation is independently correlated with size of primary tumor

Correlation of *HOPX* Q-MSP value with each clinicopathological tumor factor was analyzed (Table 2 and Supplementary Figure 2). Q-MSP value was associated with male gender, tumor diameter, extrathyroidal extension and lymph node metastasis in univariable analyses. Among them, only tumor diameter was independently correlated with Q-MS *P* value ≥ 6.1 (*p =* 0.016, Table 2).

### *HOPX* expression is correlated with genes toward inhibiting cancer cell progression and dedifferentiation

In TCGA clinical data on PTC patients (eBioPortal for Cancer Genomics https://www.cbioportal.org/) (Papillary thyroid carcinoma, Cell, 2014) [23], *HOPX* was positively correlated with *CDH6* and *LAMC2* that are involved in differentiation, while *HOPX* was negatively associated with *HHEX* and *ERBB4* that contribute to dedifferentiation. Also, *HOPX* was correlated with genes in a manner toward suppression of cancer cell progression; downregulation of *TNRC6C*, *PAX8*, *TSHR*, *DYRK1A/B*, and *SLIT3* (pro-proliferation/migration), and upregulation of *PCDH8/9*, *CDC42EP3/4/5*, and *TJP3* (anti-proliferation/migration) (Supplementary Figure 4 and Supplementary Table 2).

## DISCUSSION

We have reported that *HOPX* is frequently methylated in a cancer-specific manner, and functions as a tumor suppressor gene in esophageal, gastric, colon and pancreas cancers in the previous studies [12–15, 21, 24]. These previous results prompted us to seek the clinical ramifications of epigenetic silencing of HOPX in papillary thyroid cancer (PTC) which represents the vast majority of thyroid malignancies. Indeed, epigenetic silencing of HOPX by its promoter methylation was observed with high sensitivity and specificity in PTC as well (Figure 3). Notably, the promoter methylation of *HOPX* is independently associated with poor prognosis especially in patients with stage I PTC.

*HOPX* has unique 3 transcript variants (α, β and γ) which share the same open reading frame. Among them, only *HOPX*-β harbors CpG islands in its promoter region. The bisulfite sequencing analyses showed that the promoter methylation of *HOPX*-β in its CpG islands was observed in the 5 human thyroid cancer cell lines except MTC cell line TT. The expression of HOPX-core is faint or undetectable in human PTC cell line K1, FTC line RO82-w-1, and UTC line 8305c in consistent with the very weak or undetectable expression of *HOPX*-β without HOPX-α/γ expression (Figure 1B). In these cell lines, HOPX-β and HOPX-core expression were restored by 5-Aza-dC with/without TSA, suggesting that HOPX expression depends on HOPX-β expression. On the other hand, although the promoter methylation of *HOPX*-βwere also observed in FTC line FTC133 and UTC line 8505c, these lines express *HOPX*-core reflecting *HOPX*-α expression. Demethylation treatment of 8505c and FTC133 actually reactivated HOPX-β expression but enhancement of HOPX-core expression was not observed (data not shown). On the other hand, DLD1 (colon cancer cell line) expresses weak HOPX-α but HOPX-βreactivation significantly increased HOPX-core expression in our previous report [12]. These results suggest that HOPX-β is dominant to HOPX expression when HOPX-α expression is insufficient. These results suggest that the mechanisms of HOPX expression is different among histological types of thyroid cancer.

Consistent with our previous study in gastrointestinal cancers and breast cancer [12–14, 21], the promoter hypermethylation of *HOPX* dramatically predicts disease recurrence in patients with PTC. Actually, a very recent study reports hypermethylation of *HOPX*-β was associated with poor survival in differentiated thyroid cancer [25]. In our current study, the hypermethylation of *HOPX* independently correlated with tumor size among the tumor factors in PTC. Immunohistochemistry of Ki-67 supports that hypermethylation of *HOPX* leads to more cancer cell proliferation in patients with PTC (Table 2, Figures 5C and 5D). Of note, *HOPX* promoter hypermethylation is critically associated with poor prognosis in patients with stage I PTC but only marginally in patients with stage II. These results indicate that HOPX may particularly regulate early steps of cancer progression. Indeed, enforced expression of HOPX suppress cell proliferation, invasive activities, and anchorage-independent growth (Figure 2) that reflects metastatic potential of cancer cells [26]. In TCGA PTC data set, putative HOPX target co-expressed gene profiles support tumor suppressive function of HOPX (Supplementary Table 2 and Supplementary Figure 4). In addition, co-expressed gene profiles indicate that HOPX may suppress dedifferentiation of cancer cell in consistent with our previous study on colorectal cancer in which epigenetic silencing of HOPX was associated with poor differentiation [12].

HOPX is an unusual homeodomain-containing protein that is unable to bind DNA, and modulates chromatin structure and represses nuclear transcription [17]. *HOPX* is essential for normal cardiac growth and development, and is involved in a wide range of organ development [9, 27–30]. Moreover, HOPX exclusively specifies cardiomyoblast commitment by harnessing Wnt signialing through Smad4 interaction [31]. Indeed, poor differentiated histology is significantly associated with HOPX silencing by its promoter methylation in colorectal cancer [12, 32], and HOPX represents +4 quiescent intestinal stem cells which can interconvert between Lgr5^+^ active stem cells [33]. Recent study reported that HOPX suppresses tumor progression by an epigenetic regulation of SNAIL transcription through the enhancement of histone H3K9 deacetylation in the SNAIL promoter [34]. Moreover, HOPX plays a critical role in epithelial cell homeostasis and functions as a tumor suppressor in head and neck cancer [35]. These findings suggest that silencing of HOPX may promote dedifferentiation of epithelial cells and accelerate cancer initiation and progression. Further insights into the association of HOPX with radioactive iodine-refractoriness or chemosensitivity are needed in the next study.

## MATERIALS AND METHODS

### Cell lines and human thyroid cancer tissue samples

Human thyroid cancer cell lines, K1, TT, 8305c, 8505c, FTC-133, and RO82-w-1 were purchased from RIKEN Bio Resource Center (Tsukuba, Japan). An esophageal squamous cell carcinoma cell line TE1 and TE15 were also purchased from RIKEN Bio Resource Center as control cell lines. K1 and FTC-133 were cultured in DMEM/F12 Glutamax (GIBCO, Carlsbad, CA, USA) supplemented with 10% FBS. 8305c, 8505c, RO82-w-1, TE1, and TE15 were maintained in RPMI1640 Medium (GIBCO) supplemented with 10% FBS. TT was cultured in ATCC-formulated F12 K Medium (ATCC, Manassas, VA, USA) containing 10% FBS.

One-hundred ninety-one patients with papillary thyroid cancer (PTC) who have undergone curative surgical resection of the primary tumors at the Kitasato University Hospital between January 1, 2000 and December 31, 2006 were assessed. These patients were clinicopathologically analyzed. Independent 20 PTC patients were initially analyzed for the methylation status of HOPX-β as a study set. Clinicopathological characteristics of 191 PTC patients were shown in Table 1. TNM classification was made according to the latest 8th edition of the Unio Internationalis Contra Cancrum (UICC) staging system. This study was approved by the IRB of the Kitasato University School of Medicine (the IRB approved #B17-233), and was performed in accordance with the clinical research guidelines of the ethics committee of the Kitasato University School of Medicine. All patients gave written informed consent.

### Collection of TCGA public data

Gene expression data on 496 PTCs were downloaded from http://www.cbioportal.org/ (Papillary Thyroid Carcinoma (TCGA, Cell 2014). csv).

### Bisulfite treatment of DNA and sequencing analysis

Tissue sections from primary tumors and corresponding normal tissues were stained with hematoxylin and eosin and dissected under microscope. Genomic DNA was extracted from cell lines or formalin-fixed paraffin embedded (FFPE) tissues using a QIAamp DNA Mini Kit or a QIAamp DNA FFPE Tissue Kit (Qiagen, Hilden, Germany), respectively. Bisulfite treatment was done by using an EZ DNA Methylation-Gold TM Kit (Zymo Research, Orange, CA, USA) and the bisulfited DNA was subsequently amplified by PCR. Primer sequences were designed to recognize the DNA alterations caused by bisulfite treatment (Supplementary Table 1). The PCR products were purified with a QIAquick PCR Purification Kit (Qiagen) and were sequenced using a Big DyeR Terminator v3.1 Cycle Sequencing Kit (Applied Biosystems, Foster City, CA, USA).

### Quantitative methylation-specific PCR (Q-MSP)

TaqMan methylation-specific PCR (Q-MSP) was carried out using a iQ Supermix (Bio-Rad, Hercules, CA, USA) in triplicate on a C1000 TouchTM Thermal Cycler CFX96 Real Time System (Bio-Rad). PCR conditions, the primer and probe sequences are provided in Supplementary Table 1. Serial dilutions of bisulfite modified DNA from human esophageal cancer cell line TE1 was used to construct the calibration curve on each plate as a methylation positive control, and TE15 served as a methylation negative control as reported [14]. The methylation value was defined by a ratio of amplified signal value of methylated *HOPX* normalized to β-actin and then multiplied by 100 (Q-MSP value).

### RNA purification and reverse transcription-polymerase chain reaction (RT-PCR)

Total RNA from cell lines was extracted using an RNeasy Mini Kit (Qiagen), and reverse-transcribed with a SuperScript III reverse transcriptase kit (Invitrogen, Carlsbad, CA, USA). Primer sequences are described in Supplementary Table 1. RT-PCR was performed, and the PCR products were separated on 1.5% agarose gel, then visualized by EtBr. β-actin served as an internal control.

### Western blot analysis

Whole cell lysates were extracted in RIPA buffer (Pierce, Rockford, IL, USA) supplemented with a Halt Protease Inhibitor Cocktail kit (Pierce) and a Halt Phosphatase Inhibitor Cocktail kit (Pierce). The protein concentrations were determined using a Coomassie Plus-The Better Bradford Assay kit (Pierce), separated on NuPAGE 4–12% Bis–Tris Gel (Invitrogen). The mouse HOPX monoclonal IgG_1κ_ antibody (3D6; Sigma-Aldrich, Inc, St Louis, MO, USA), and mouse β-actin IgG_2a_ monoclonal antibody (Sigma-Aldrich) were used. A WesternBreeze® Chemiluminescent Kit–Anti-Mouse (Invitrogen) was used for detection.

### 5-aza-2’-deoxycytidine and trichostatin A treatments in human breast cancer cell lines

Cells (1 × 10^6^ cells/T-75 flask) were treated with 1 or 5μM of a demethylating agent 5-aza-2′-deoxycytidine (5-Aza-dC) (Sigma-Aldrich, St Louis, MO, USA) dissolved in 50% acetic acid (Wako, Osaka, Japan) or mock-treatment with PBS (GIBCO) including the same amount of acetic acid every 24 h for 4 days. When combined with a histone deacetylase (HDAC) inhibitor trichostatin A (TSA) (Sigma-Aldrich), 300 nM of TSA was added to the medium for the final 24 h.

### Immunohistochemistry

Primary PTC tissue and the adjacent normal thyroid tissue were fixed in formalin and embedded in paraffin. Four mm thick serial sections were made. Briefly, antigen retrieval was performed by autoclaving for 10 min at 120° C in antigen retrieval buffer (1.8 mM citric acid, 8.2 mM sodium citrate, 0.05% Tween 20, pH 6.0) for HOPX. For Ki-67, antigen retrieval was performed with microwave for 5 min, three times in 1mM ethylenediaminetetraacetic acid buffer. Endogenous peroxidase activity was blocked by incubation in 3% H_2_O_2_/methanol at room temperature for 5 min, and non-specific binding was blocked by incubation with 1% diluted normal horse serum for 30 min. Either anti-HOPX mouse IgG_1κ_ monoclonal antibody (3D6, Sigma) (1:100 dilution) or anti-Ki-67 antibody (MIB-1, DAKO) (1:300 dilution) was added and the slices were incubated at 4° C overnight. For HOPX, immune complexes were detected with a Vectastain Elite ABC kit (Vector Laboratories, Inc, Burlingame, CA) according to the manufacturer's instruction. For Ki-67, immune complexes were detected with a Histofine Simplestain Max PO (Nichirei) according to the manufacturer's instruction. These immune complexes were detected using the 3,3′-diaminobenzidine (DAB) substrate as a chromogen for 2 min (HOPX) or 30sec (Ki-67). Sections were counterstained with hematoxylin. HOPX expression level was graded according to the diagnostic criteria of American Society of Clinical Oncology/College of American Pathology 2007 guidelines as follows [36]. IHC 1+: light staining of more than 10% of the specimens; IHC 2+: moderate staining of more than 10% and less than or equal to 30% of the specimens; IHC3+: strong staining of more than 30% of the specimens. Ki67 intensity was classified as follows. IHC 1+: light staining of less than 1% of the tumor cells; IHC 2+: moderate staining of more than or equal to 1% and less than 5% of the tumor cells; IHC3+: strong staining of more than or equal to 5% of the tumor cells.

### Plasmid and transfection

A full-length cDNA of HOPX was previously isolated and subcloned into pcDNA^TM^3.1D/V5-His-TOPO vector (pcDNA^TM^3.1-HOPX) [12]. The vector with self-ligation was used as a control. Plasmid vectors were transfected into PTC cell line K1 using a Lipofectamine 2000 reagent (Invitrogen).

### Proliferation assay

Cell proliferation and viability (2 × 10^3^ cells/well) were assessed using a Premix WST-1 Cell Proliferation Assay System (Takara Bio, Tokyo, Japan) in 96-well plates. Data are expressed as an absorbance at 450 nm. Experiments were performed in triplicates.

### Matrigel invasion assay

Matrigel invasion assay was analyzed using a CytoSelect™ 24-Well Cell Invasion Assay (Cell Biolabs, Inc. San Diego, CA, USA) according to the manufacturer’s protocol. Cells were seeded at a density of 3 × 10^6^ per well with 300μL of serum free medium to an ECM-coated well (Cell Biolabs, Inc.). 10%FBS was used as a chemoattractant. After incubation for 24 hours at 37° C, the membrane of the upper chamber was incubated a clean well containing 225μL of Cell Detachment Solution (Cell Biolabs, Inc.) for 30 minutes at 37° C. Then, 1μL of 4× Lysis Buffer and 74μL of CyQuant® GR dye (Cell Biolabs, Inc.) were added to each well. After 20 minutes incubation at room temperature, 200μL of the mixture was transferred to a 96-well plate for reading fluorescence with a Varioskan LUX (Thermo Fisher Scientific Inc. Waltham, MA, USA) at 480nm/520nm. Each experiment was done in triplicate. Simultaneously, an equal number of cells were seeded on 24-well plates and incubated for 24 hours, and WST assay was performed.

### Anchorage-independent colony formation assay

Anchorage-independent cell growth was analyzed by plating 0.36% top agarose (Bacto^TM^ Ager, Becton Dickison and Company, Franklin Lakes, NL) containing 1 × 10^5^ cells on a surface of 0.72% bottom agarose in 6-well plates. Cells were fed weekly by overlying fresh soft agar solution containing G418. Colonies were visualized with EtBr after 3 wk of incubation. Two independent experiments were conducted and each experiment was done in triplicate.

### Statistical analysis

For continuous variables, Student’s *t*-test or ANOVA followed by Bonferroni test were used for comparison between two groups, or among multiple variables, respectively. c^2^ test or Fisher exact test was used for categorical variables. Clinicopathological characteristics and follow-up data were analyzed in terms of relapse free survival (RFS). The follow up time was calculated from the date of surgery to death or end-point, and patients with other disease deaths were censored. RFS was estimated by a Kaplan–Meier method, and compared using a log-rank test. Variables suggested potential prognostic factors on univariable analyses (*p <* 0.05) were subjected to a multivariable analysis using a Cox proportional-hazards model. Correlation of clinicopathological characteristics with *HOPX* Q-MSP value were also evaluated. Variables with significant correlation in univariable analyses were subjected to a multivariable logistic regression analysis. *P <* 0.05 was considered significant. All statistical analyses were conducted with JMP Pro14 (SAS Institute, Cary, NC, USA).

## CONCLUSIONS

*HOPX* promoter methylation is frequent and cancer-specific in papillary thyroid cancer (PTC). *HOPX* promoter methylation critically predicts disease recurrence particularly in stage I PTC. Furthermore, HOPX is involved in multiple functions of cancer cell progression. Therefore, silencing of HOPX expression may have significant potential as a useful biomarker in patients with PTC. Further insights into the detailed mechanism of HOPX silencing in cancer progression are likely to provide a clue to tackle PTC with treatment refractoriness.

## SUPPLEMENTARY MATERIALS


