# Supplementary material for: Epigenetic silencing of HOPX is critically involved in aggressive phenotypes and patient prognosis in papillary thyroid cancer
Source: Oncotarget. 2019 Oct 15;10(57):5906–18. doi: 10.18632/oncotarget.27187 (PMC6800262; doi:10.18632/oncotarget.27187)
Supplement: Supplementary file 1 [file oncotarget-10-5906-s001.pdf]

Epigenetic silencing of HOPX is critically involved in aggressive phenotypes and patient prognosis in papillary thyroid cancer

SUPPLEMENTARY MATERIALS

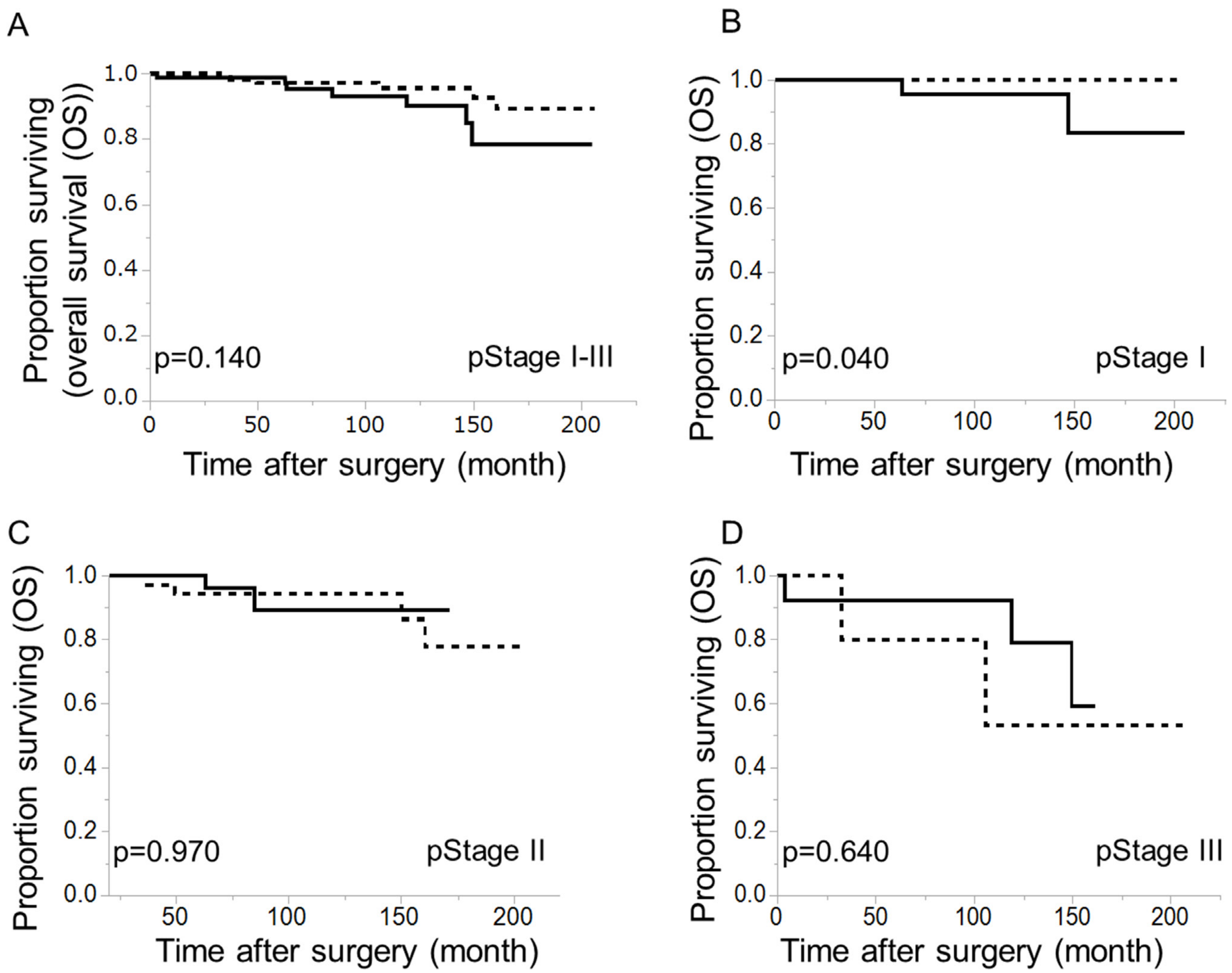

Supplementary Figure 1: Kaplan–Meier analysis of 10-year OS according to HOPX-β Q-MSP values in patients with stage I-III (**A**), stage I (**B**), stage II (**C**), and stage III (**D**). Patients with high Q-MSP values showed significantly worse OS in stage I ($p = 0.040$).

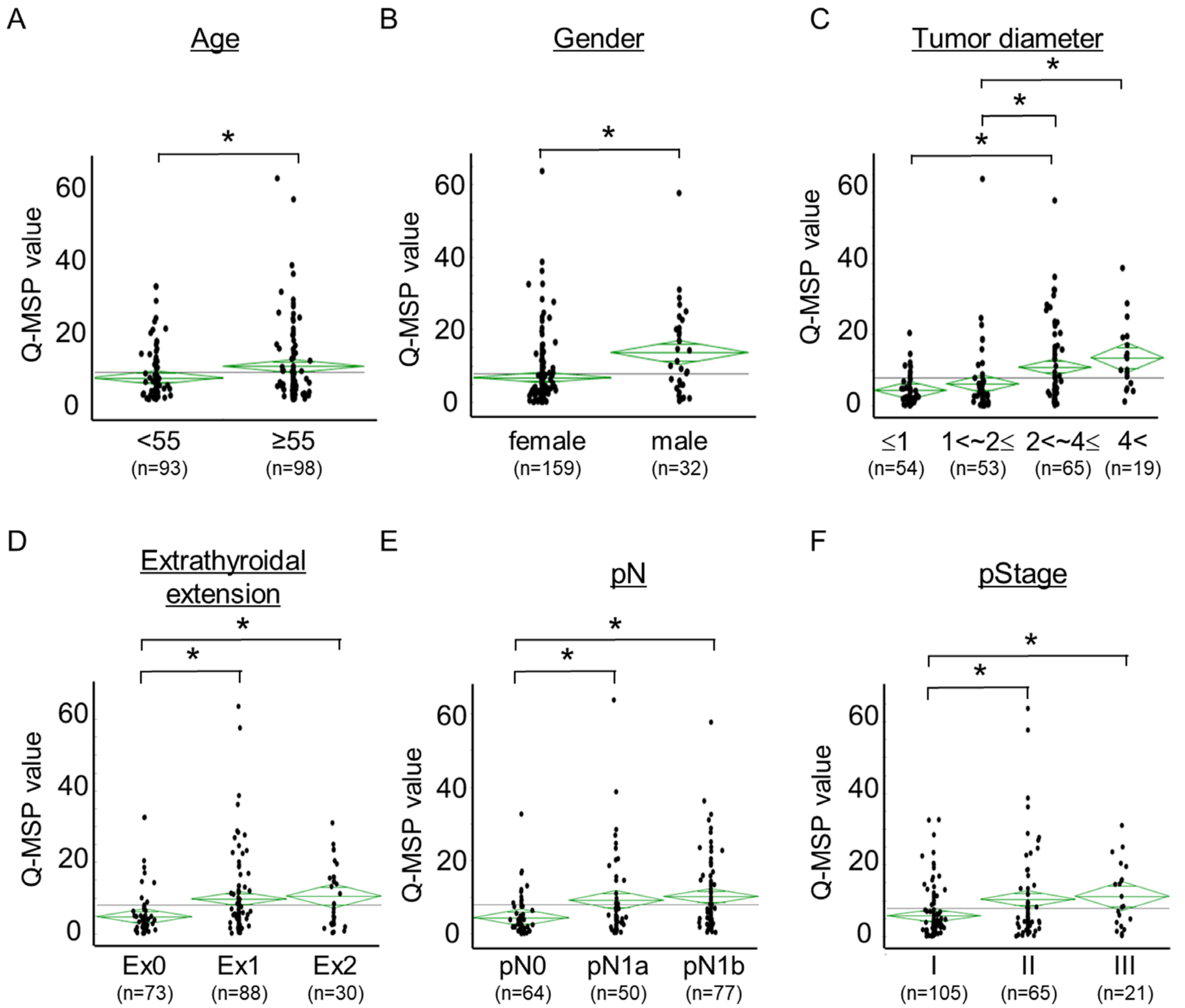

Supplementary Figure 2: Scatterplots of HOPX-β Q-MSP values of primary PTC tissues taking each tumor factors into account. *$p < 0.05$.

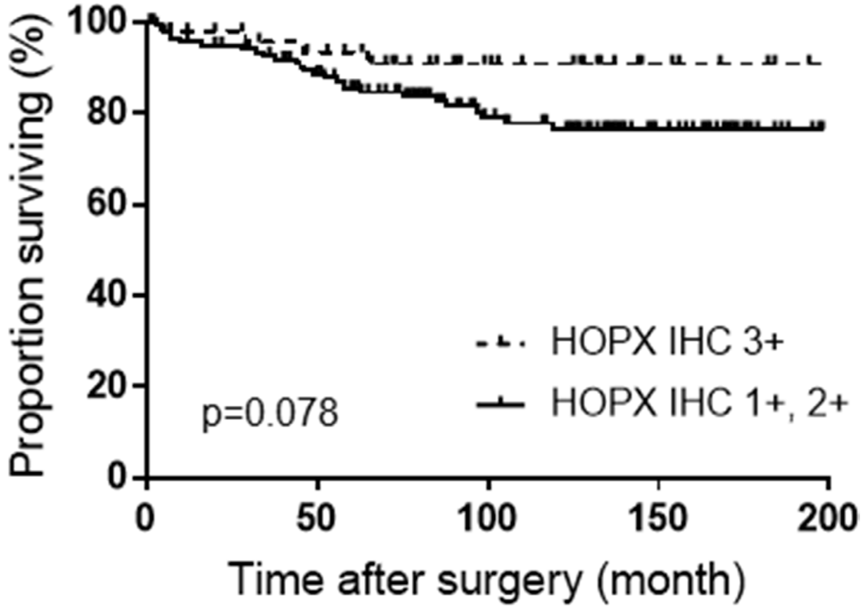

Supplementary Figure 3: Kaplan–Meier analysis of 10-year RFS according to HOPX expression in immunohistochemistry.

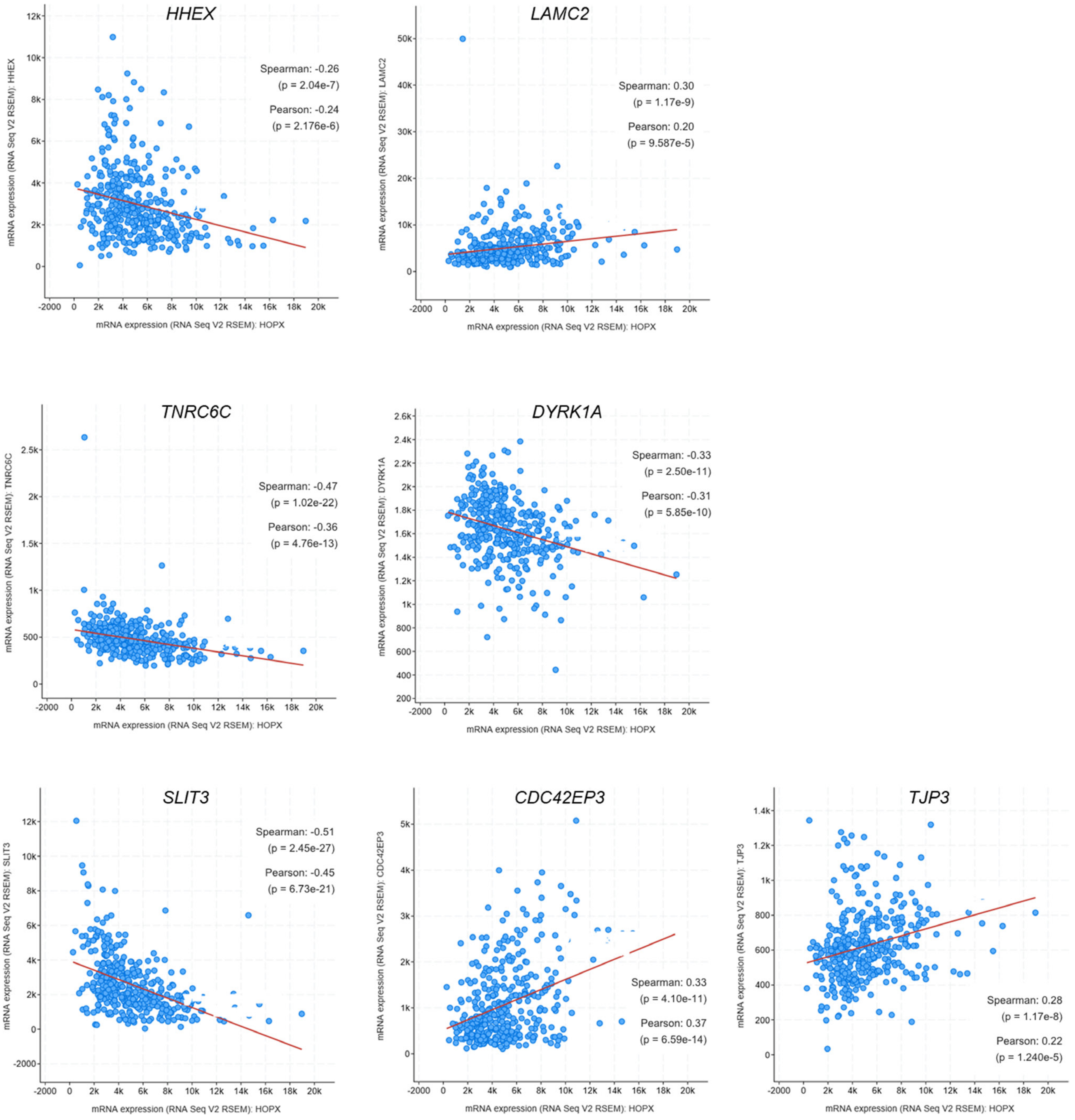

Supplementary Figure 4: Putative *HOPX* target genes whose mRNA levels were significantly correlated with those of *HOPX* in 496 PTCs of TCGA data set (http://www.cbioportal.org/) (Cell, 2014). Levels of mRNA for *HHEX*, *LAMC2*, *TNRC6C*, *DYRK1A*, *SLIT3*, *CDC42EP3,* and *TJP3* are plotted against *HOPX* mRNA levels.

Supplementary Table 1: Sequences of primers and probes for PCR

Method	Gene	Forward primer (5′>3′)	Fluorescent (5′>3′)	Reverse primer (5′>3′)
Bisulfite sequencing	*HOPX-β*	TAGTTTTGTTTGGAGAGGGTTTTAAAG		AACCTCCCCTAAAAACAAACTTAAC
TaqMan-Q-MSP	*HOPX-β*	TTTGGAGAGGGTTTTAAAGCG	CGGAGATAGAAGGTCGTTTATCGGGG AGGTCG	AACAAACTTAACAAATCGCGAA
TaqMan-Q-MSP	*β-actin*	TGGTGATGGAGGAGGTTTAGTAAGT	ACCACCACCCAACACACAATAA CAAACACA	AACCAATAAAACCTACTCCTCCCTTAA
RT-PCR/qRT-PCR	*HOPX-α and γ*	CAAACCCAGGGCTTGCGCTT		GCGGAGGAGAGAAACAGAGAT
RT-PCR/qRT-PCR	*HOPX-β*	GGTCCCCCTTTCGGGAGGAA		GCGGAGGAGAGAAACAGAGAT
RT-PCR/qRT-PCR	*HOPX-core*	CAGAGGACCAGGTGGAAATCC		GCGGAGGAGAGAAACAGAGAT
RT-PCR/qRT-PCR	*β-actin*	TCACCCACACTGTGCCCATCTACGA		CAGCGGAACCGCTCATTGCCAATGG

RT-PCR was done at 95° C for 3 min followed by 30 cycles at 95° C for 1 min, 60° C for 1 min, 72° C for 1 min, and final extension at 72° C for 10 min. 1 µl dNTP mixture, 1.5 µl MgCl2 , 0.2 µmol/l each primer and 0.2 µl Platinum Taq DNA polymerase.
Q-MSP was done at 95° C for 3 min followed by 40 cycles at 95° C for 20 sec, 60° C for 30 sec, and 72° C for 30 sec, in a 25 µl reaction volume containing 200 nmol/l fluorescect probe, and 25 µl iQTM supermix.

Supplementary Table 2: Correlated gene expression with HOPX in TCGA PTC (2014)

Correlated gene	Function	Spearman	
		r	*p*
HHEX	dedifferentiation	−0.260	<0.001
ERBB4	dedifferentiation	−0.123	0.016
CDH6	differentiation	0.131	<0.001
LAMC2	differentiation	0.303	<0.001
TNRC6C	proliferation	−0.470	<0.001
PAX8	proliferation	−0.157	<0.001
DYRK1A	proliferation	−0.330	<0.001
DYRK1B	proliferation, cell cycle	−0.126	0.013
PCDH9	suprresion of cell proliferation, tumor cell arrest at G0/G1	0.147	<0.001
SLIT3	migration	−0.512	<0.001
PCDH8	cell adhesion, suppression of migration	0.258	<0.001
CDC42EP3	regulate actin cytoskelton	0.327	<0.001
CDC42EP4	regulate actin cytoskelton	0.205	<0.001
CDC42EP5	regulate actin cytoskelton	0.196	<0.001
TJP3	linkage between the actin cytoskelton and tight-junctions	0.284	<0.001
